# Riboswitches for Controlled Expression of Therapeutic Transgenes Delivered by Adeno-Associated Viral Vectors

**DOI:** 10.3390/ph14060554

**Published:** 2021-06-10

**Authors:** Zachary J. Tickner, Michael Farzan

**Affiliations:** 1 Department of Immunology and Microbiology, the Scripps Research Institute, Jupiter, FL 33458, USA; mfarzan@scripps.edu; 2 Emmune, Inc., Jupiter, FL 33458, USA

**Keywords:** adeno-associated virus, gene therapy, transgene, aptamer, riboswitch, ribozyme, aptazyme, gene expression control, gene regulation

## Abstract

Vectors developed from adeno-associated virus (AAV) are powerful tools for in vivo transgene delivery in both humans and animal models, and several AAV-delivered gene therapies are currently approved for clinical use. However, AAV-mediated gene therapy still faces several challenges, including limited vector packaging capacity and the need for a safe, effective method for controlling transgene expression during and after delivery. Riboswitches, RNA elements which control gene expression in response to ligand binding, are attractive candidates for regulating expression of AAV-delivered transgene therapeutics because of their small genomic footprints and non-immunogenicity compared to protein-based expression control systems. In addition, the ligand-sensing aptamer domains of many riboswitches can be exchanged in a modular fashion to allow regulation by a variety of small molecules, proteins, and oligonucleotides. Riboswitches have been used to regulate AAV-delivered transgene therapeutics in animal models, and recently developed screening and selection methods allow rapid isolation of riboswitches with novel ligands and improved performance in mammalian cells. This review discusses the advantages of riboswitches in the context of AAV-delivered gene therapy, the subsets of riboswitch mechanisms which have been shown to function in human cells and animal models, recent progress in riboswitch isolation and optimization, and several examples of AAV-delivered therapeutic systems which might be improved by riboswitch regulation.

## 1. Introduction

One of the major barriers to human gene therapy is safe, efficient delivery of genetic material and/or editing complexes to specific tissues or cell types. Lipid nanoparticles (LNPs) are immunogenic, provide only transient expression, and can be effectively administered through intramuscular injection, making them ideal vectors for transgene therapeutics such as mRNA vaccines [1]. However, for therapies which require systemic administration, tissue targeting, and/or long-term expression to improve efficacy or reduce toxicity, adeno-associated virus (AAV) vectors are preferred [2]. AAV is a small, replication-deficient parvovirus first identified as a contaminant in adenovirus cultures in 1965 [3]. AAV is much less immunogenic than other viruses, and vectors can be engineered both to promote and to suppress integration into the host genome [4,5,6]. AAV serotypes exhibit various tissue tropisms [7], and new capsid variants can be designed or selected for even greater cell type specificity [8,9]. AAV possesses a ssDNA genome which must normally be converted to dsDNA for efficient nuclear localization and gene expression, but engineered self-complementary AAV (scAAV) genomes bypass the need for second-strand synthesis and exhibit extremely efficient transduction [10]. Furthermore transduction-competent virions can be generated even after 96% of the native genome has been replaced, leaving room for a transgene expression cassette. This also leaves only short inverted terminal repeats (ITRs) necessary for packaging and nuclear localization, rendering the virus completely replication deficient and severely limiting integration into the host genome [5]. Regardless of these advantages, the small size of the AAV genome can present a challenge: AAV vectors can only package and deliver transgenes up to 4.7 kb in size, while this is reduced to 2.4 kb in scAAV [11]. Strategies have been developed for delivery of large transgenes, but the simplest method is to minimize accessory elements such as promoters to maximize “headspace” for transgene insertion [12,13]. Following nuclear translocation, ITR-mediated concatemerization of AAV genomes can produce circular episomes which provide long-term transgene expression even in the absence of integration [14]. These properties combine to make AAV an excellent tool for enabling specific, long-term transgene expression, and several AAV-based gene therapies are currently approved for use in Europe and the United States [15].

A second barrier to human gene therapy is ensuring appropriate levels of transgene expression. Tissue-tropic AAV and cell type-specific promoters or miRNA target sites can help to prevent off-target expression, but regulation is still required to achieve therapeutic levels of, or to avoid deleterious immune responses to, the transgene product. There are several systems which enable exogenous control of gene expression: these include the Tet-On and Tet-Off systems which enable strong induction or inhibition of transgene expression in response to the small-molecule drug doxycycline, optogenetics approaches which allow highly specific spatial and temporal control of transgene expression using light, and even systems which control transgene expression using sound [16,17,18,19,20]. Several of these systems have been used to regulate therapeutic transgenes in animal models, but they rely upon expression of non-mammalian proteins to function; in addition to being immunogenic, inclusion of the genes coding for regulatory proteins also occupies precious space in the AAV genome. In addition to controlling dosing, suppression of transgene expression can also improve yields during AAV vector production [21] and could help to prevent anti-transgene immune responses during heightened immune surveillance following AAV administration [22].

Riboswitches are structured nucleic acids which regulate gene expression in response to ligand binding. Riboswitches are small (often < 100 nt), can function independently of proteins, and are non-immunogenic, therefore occupying less vector headspace and presenting less risk in therapeutic applications [23]. Riboswitches consist of a ligand-sensing aptamer domain joined to an expression platform which regulates gene expression in response to aptamer binding. Aptamers were first reported in 1990, when RNA aptamers to protein and small-molecule targets were isolated through in vitro selection method known as systematic evolution of ligands by exponential enrichment (SELEX) [24,25]. Meanwhile, the 1989 Nobel prize was awarded to Thomas Cech for the discovery of ribozymes (catalytic RNAs), and high-specificity, high-affinity ligand binding by RNA was proposed as a possible mechanism of allostery in the “RNA world” hypothesis [26,27,28]. In 1997 Tang and Breaker united these RNA devices in a synthetic riboswitch in which an in vitro selected, ATP-binding aptamer was combined with a self-cleaving hammerhead ribozyme expression platform to allow ligand-regulated control of RNA stability in vitro [29]. The in vitro selected theophylline, tetracycline, and guanine aptamers have also been incorporated into a variety of rationally-designed riboswitches [30]. Natural riboswitches were first reported in 2002, when the Breaker group identified several RNA motifs which regulated bacterial gene expression in response to binding by small molecule metabolites [31,32,33]. The vast majority of natural riboswitches occur in bacteria, with only a limited number of thiamine pyrophosphate (TPP) riboswitches occurring in eukaryotes such as plants and fungi along with possible examples in viruses [34,35,36]. To date, no riboswitches have been identified in mammals, although protein-directed RNA switches serve similar functions [37]. Over 40 classes of bacterial riboswitch have been discovered, and high-throughput sequencing and analysis pipelines have been developed to speed their discovery [38]. Meanwhile, techniques have been designed for automated design of riboswitches [39], as well as for riboswitch selection in vitro [40,41,42], in bacteria [43,44,45,46,47], and in yeast [48].

Several riboswitches have been shown to regulate gene expression in mammalian cells, although improving their performance in vivo presents a continuing challenge. Riboswitches operating in mammalian cells have been recently reviewed by Yokobayashi, but many exciting new advances in therapeutic riboswitch development have occurred in the intervening three years [23]. This review presents the mechanisms of several riboswitches with therapeutic potential, their performance in mammalian cells and animal models, and recent progress in improving their regulatory properties and developing methods for riboswitch screens and selections. Several recent publications have also presented methods for screening and selecting novel riboswitches specifically for function in human cells, representing significant progress in the identification of new therapeutic transgene regulators. Finally, several potential therapeutic applications of riboswitches are discussed.

## 2. Riboswitch Regulation of Transgene Expression in Mammals

Riboswitch regulatory or dynamic ranges are determined by the difference in expression between the ligand unbound state (basal expression) and the ligand bound state (induced/suppressed expression). Success as a regulator thus depends not only on the regulatory range, but also on whether expression levels in these two states are appropriate for the intended application. Riboswitches can be tuned or selected for improved function in one or more cell types, and components can frequently be exchanged to generate novel riboswitches which function in bacterial systems [49,50,51,52,53]. However, both natural and synthetic riboswitches often perform poorly in eukaryotic (particularly mammalian) cells [54]. The bacterial cytosol and most in vitro aptamer selection environments contain higher concentrations of Mg^2+^ (an essential ion for RNA folding) compared to human cells, while in vitro selection conditions also struggle to simulate cellular processes such as ion chelation and molecular crowding [55,56,57]. Eukaryotes also possess distinct sets of polymerases, RNA modifying enzymes, RNA-binding proteins, folding chaperones, and nucleases [58,59,60]; some riboswitches incorporate aptamers which can fold and bind ligands in eukaryotic cells, but use expression platforms based on prokaryote-specific mechanisms such as rho-independent transcription termination [53,61,62,63]. Even for switches which do function in eukaryotes, expression control in mammalian cells can be particularly challenging. For example, placement of aptamers in the 5′ UTR of an mRNA enables efficient ligand-induced translational repression in multiple eukaryotic species, but is much less effective in mammals. Despite these challenges, several riboswitches have been shown to function in mammalian cells [23]. The ligands, regulatory ranges and mechanisms of these switches are discussed below and are summarized in Table 1.

### 2.1. Riboswitches Regulating mRNA Processing

**Figure 1:**
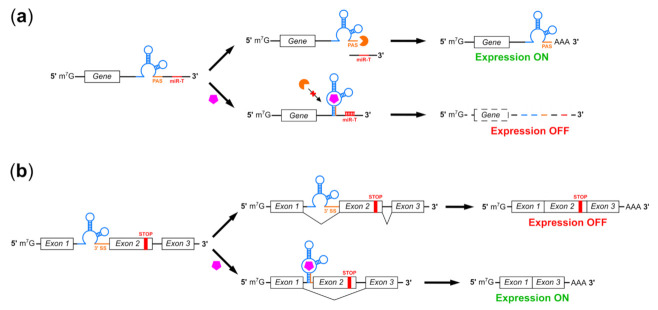
Riboswitch Regulation of mRNA Processing in Mammalian Cells. (**a**) Regulation of polyadenylation. In the absence of ligand, the polyadenylation site (PAS, orange) is bound by the polyadenylation complex (orange), which removes a downstream miRNA target site (miR-T, red) and adds a poly-A tail to enable expression. Ligand binding (purple) to an aptamer domain (blue) sequesters the PAS, blocking processing and promoting mRNA degradation by exonucleases and miRNA-induced silencing [66]. (**b**) Regulation of splicing by ligand-induced exon skipping. In the absence of ligand binding, an exon with a premature stop codon is spliced into the mRNA, preventing gene expression. Ligand binding sequesters spliceosome recognition elements such as the 3′ acceptor site (3′ SS, orange), promoting exon skipping and expression of a full-length, functional protein [68,69,70,71].

Many bacterial riboswitches operate at the transcriptional level, but differences in transcription mechanisms and greater compartmentalization of transcription and translation present unique challenges in eukaryotic systems [64]. Common bacterial riboswitch mechanisms such as rho-independent termination are ineffective in eukaryotes, although components of bacterial riboswitches have been adapted for use in mammalian cells [65]. Several groups have developed riboswitches which regulate eukaryote-specific steps in mRNA processing (Figure 1). A notable example is provided in a recent publication by Spöring et al., who reported 5.2-fold suppression of reporter gene expression in human cells mediated by ligand-induced sequestration of the eukaryotic polyadenylation signal (Figure 1a) [66]. This switch was combined synergistically with other regulators such as miRNAs or aptazyme riboswitches to achieve higher regulatory ranges. Addition of a self-cleaving riboswitch produced guanine-mediated, 24-fold suppression of gene expression in human cells, compared to 9.8-fold suppression for the self-cleaving switch alone. However, the majority of switches which control mRNA processing in eukaryotes, including natural TPP riboswitches in plants and fungi, target mRNA splicing [67].

Eukaryotic TPP riboswitches inspired multiple groups to design synthetic riboswitches which used in vitro selected aptamers to regulate pre-mRNA splicing (Figure 1b). Kim et al. used the theophylline aptamer to control accessibility of 3′ splice sites and branchpoints, with both types of switch demonstrating control over splicing in HeLa nuclear extracts and branchpoint switches enabling a modest (~2-fold) increase in exon skipping in HeLa cells [68]. Weigand and Suess used the tetracycline aptamer to control accessibility of the 5′ splice site, achieving 32-fold suppression of reporter gene expression in live yeast treated with 250 µM tetracycline [69]. In 2018 Vogel et al. combined elements of both approaches to control 3′ splice site accessibility in HeLa cells using the tetracycline aptamer [70]. The authors demonstrated 5.7-fold induction of reporter gene expression in response to tetracycline when aptamer binding promoted skipping of a short exon containing a premature stop codon; combining this switch with a tetracycline-suppressible self-cleaving ribozyme in the 3′ UTR increased this to 7-fold induction. The authors also used this system to regulate CD20 expression, demonstrating tetracycline-regulated cell killing by a therapeutic antibody. A recent report by Finke et al. describes similar on-switches in which the tetracycline aptamer controls 5′ splice site accessibility and provides 16.9-fold induction of transgene expression in HeLa cells and over 20-fold induction in *C. elegans* [71]. In addition to possessing a higher regulatory range, these switches can also be engineered to place a premature stop codon within the tetracycline aptamer itself, obviating the need for an additional alternative exon sequence and producing a smaller (~60 nt) switch more suitable for use in AAV.

Protein-binding aptamers have also been used to regulate splicing events. Culler et al. used aptamers to endogenous proteins such as NF-κB p50 and β-catenin to enable regulation of alternative splicing in response to cellular signaling events [72]. Switches based on this mechanism promoted 2-4-fold suppression of gene expression in response to signaling molecules such as TNF-α or LTD4. The bacterial TetR protein has also been adapted for use in splicing regulation, with TetR aptamers providing tetracycline-mediated control over splice site accessibility and allowing regulation of gene expression in human cells [73]. As with other non-self protein-mediated expression control systems however, TetR immunogenicity and the size of its expression cassette may limit use in AAV. Even so, the recent use of this system by Mol et al. to control inclusion of an alternative exon with a nuclear localization sequence instead of a premature stop codon points toward a wider array of applications for riboswitches which mediate splicing [74]; combining riboswitches with orthogonal ligands could be used to control both expression and function of transgene products.

Riboswitches have also been used to control non-canonical splicing mechanisms in mammalian cells. In 2014, Kim et al. reported allosteric control of trans-splicing ribozymes which could regulate both endogenous and transgene expression [75]. The authors had previously adapted the *Tetrahymena* group I intron to splice exogenous 3′ sequences into pathogenic mRNAs in human cells and mouse cancer models [76,77]. Replacement of multiple stem-loops with theophylline aptamers yielded ribozymes which were activated by theophylline binding, and inclusion of a short complimentary sequence targeted these constructs to mRNAs encoding an oncogene. Addition of theophylline promoted group I intron-mediated exchange of the oncogene-coding region for a transgene enhancing ganciclovir-mediated cytotoxicity, enabling inducible cell killing specifically in cells expressing the oncogene. The ability of a single, relatively compact switch to regulate both transgene and endogenous gene expression makes this mechanism an attractive candidate for use in multifunctional AAV therapeutics.

### 2.2. Riboswitches Controlling Translation Initiation

**Figure 2:**
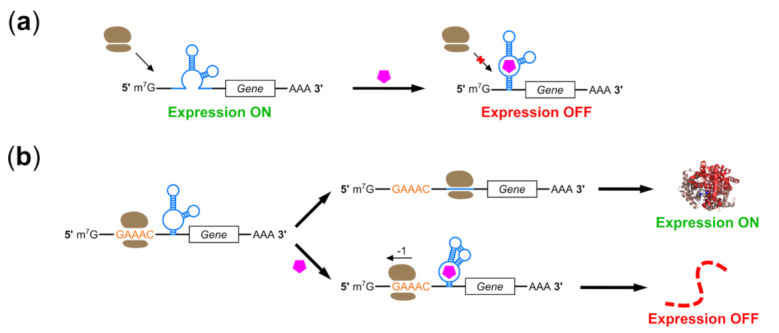
Riboswitches Regulating Translation Initiation and Elongation in Mammalian Cells. (**a**) Translation roadblock. Binding of a ligand (magenta) stabilizes the structure of an aptamer (blue) in the 5′ UTR, inhibiting translation complex assembly or procession [79,85,86]. This approach is less effective in mammalian cells than in bacteria or yeast, possibly due to differences in ribosome binding or 40S ribosomal subunit scanning [54,80,83,84]. This mechanism can also be mediated by ligand-responsive oligonucleotides binding in trans [89]. (**b**) Programmed -1 ribosomal frameshifting (-1 PRF). In the absence of ligand binding, ribosomes proceed efficiently through -1 PRF sequences and synthesize functional, in-frame protein products. Ligand binding stabilizes a structured RNA stimulator containing an aptamer (blue), causing -1 ribosomal frameshifting on an upstream slippery sequence (orange) and promoting premature termination or expression of a non-functional, -1 frameshifted protein [106,108,109,110].

As noted above, switches which block initiation by placing aptamers in the 5′ UTR of an mRNA face unique challenges in eukaryotic, and particularly mammalian cells. For example, Ogawa notes that initiation involves ribosome loading onto the internal Shine–Dalgarno sequence in prokaryotes but onto the 5′ terminus in eukaryotes, limiting options for aptamer placement and complicating on-switch development [78]. However, several switches have been developed which function in mammals using this “roadblock” mechanism (Figure 2a). In 1998, Werstuck et al. reported 10-fold suppression of reporter gene expression in CHO cells by placing an aptamer sequence in the 5′ UTR of an mRNA; however, these regulatory ranges were achieved by treating cells with millimolar concentrations of Hoechst dye derivatives chosen for cell permeability [79]. Switches regulated by well-tolerated, FDA-approved therapeutics such as theophylline and tetracycline have enabled expression control in yeast, wheat germ extract, and *X. laevi* oocytes through disruption of scanning by the 40S ribosomal subunit, but these were either not tested in mammalian cells or showed reduced performance in mammalian cells and lysates [80,81,82]. Differences in position-dependent effects of structured RNAs in the 5′ UTR, differences in 5′ cap recognition, and/or the greater ability of mammalian 40S subunits to scan through structured RNA have all been suggested as possible explanations [54,80,83,84].

These obstacles remain relevant even with significant advances in riboswitch screening and selection technology. In 2018, Groher et al. used conventional SELEX to isolate aptamers to ciprofloxacin (CFX), inserted them into the 5′ UTR of a constitutively-expressed GFP gene in yeast using homologous recombination, and screened thousands of constructs for in vivo riboswitch activity [85]. This selection and screening method rapidly isolated novel CFX aptamers and riboswitches which could suppress gene expression 7.5-fold in yeast; however, when transferred to HeLa cells, the same switches only achieved 1.8-fold regulation in response to 250 µM CFX despite the aptamer forming a large (<100 nt) pseudoknot structure. This poor performance compared to the Hoechst dye aptamer switch is interesting; the CFX aptamer is approximately 30 nt longer than the Hoechst dye aptamer, but binds a smaller ligand and assumes a pseudoknot rather than a hairpin structure. Cell permeability of these ligands may also help to explain these results. A follow-up publication used a similar selection-and-screening strategy to identify paromomycin-mediated switches, replacing conventional SELEX with capture-SELEX to favor enrichment of aptamers with riboswitching capability [86]. The enriched aptamers provide 8.5-fold regulation in yeast, but the authors do not report results for mammalian cells. Goldfless et al. also used a combination of selection and rational design to develop aptamers which provided tetracycline-mediated induction of initiation when localized to the 5′ UTR in yeast [87]. However, this was achieved by using aptamers which bound TetR in the absence of tetracycline. While protein binding may provide an excellent roadblock, the need for coexpression of an immunogenic protein makes these switches poorly suited for use in AAV-mediated therapies.

The roadblock mechanism can also be implemented by small molecule-regulated, 5′-UTR-complementary oligonucleotides. Oligonucleotides complementary to the 5′ UTR provide both a bulky ligand and a base paired structure as obstacles to initiation without the need for exogenous protein expression, and several groups have used aptamers to control annealing of such trans-acting regulatory RNAs. In 2005, Bayer and Smolke designed regulator RNAs in which binding-induced strand exchange exposed a sequestered sequence complementary to the 5′ UTR and start codon of an mRNA [88]. These so-called “antiswitches” functioned in yeast but were ineffective in mammals. More recently, Liu et al. reported a successful application of this strategy in human cells [89]. Rather than using aptamers to control hybridization of regulator RNAs, the authors designed short RNAs which hybridize constitutively to sequences in the 5′ UTR or protein-coding region of a reporter transgene. Hybridization alone does not inhibit expression, reflecting the high bar for physical obstruction of the mammalian ribosome. However, attachment of two aptamers to the complementary oligonucleotide enabled approximately 10-fold suppression of transgene expression in HEK293 cells by tetracycline or theophylline. These switches were most effective when targeted to the 5′ UTR and a single aptamer provided only weak regulation while three aptamers did not significantly improve regulatory range, shedding light on the requirements of the “roadblock” mechanism in mammals. The authors also illustrate 15-fold activation of gene expression when small molecule-binding aptamers are replaced by aptamers to the initiation factor eIF4G [90]. These latter constructs are not riboswitches per se, but incorporation of small molecule-binding aptamers which regulate eIF4G aptamer binding might yield ligand-regulated on-switches. The small size and modular design of these switches with respect to both target gene and ligand make them attractive targets for use with AAV vectors.

### 2.3. Riboswitches Controlling Alternative Initiation Mechanisms

Several groups have also developed switches for regulating alternative initiation mechanisms in eukaryotes. In 2011, Ogawa used rational design to generate switches in which aptamers to theophylline, tetracycline, flavin mononucleotide, and sulforhodamine B were all able to regulate internal ribosome entry site (IRES)-mediated transgene expression [78]. The author demonstrated that inclusion of an anti-IRES (aIRES) sequence could disrupt IRES folding and suppress translation, while a complementary anti-anti-IRES (aaIRES) sequence could sequester the aIRES and restore translation. Placement of aptamers adjacent to the aaIRES and a modulator sequence allowed ligand-dependent aaIRES exposure through a strand exchange mechanism, promoting sequestration of aIRES and thus proper IRES folding and initiation. This system enabled over 30-fold induction of eukaryotic gene expression. These constructs were later adapted into IRES-mediated off-switches [91], including off-switches in which IRES stem formation was controlled directly by aptamer binding rather than by binding-induced strand exchange with aIRES or aaIRES [92]. A 2020 follow-up publication reported several similar switches which were isolated using a strategy similar to that used to select paromomycin aptamers [86,93]. The authors first isolated aptamers to a 6 nt, single-stranded “nano-DNA” (nDNA) using a SELEX method designed to mimic the target environment and to promote stem formation upon ligand binding, integrated these aptamers into aIRES/aaIRES-containing switches, and achieved 25-fold activation of gene expression in response to micromolar concentrations of nDNA. Unfortunately all results for these constructs were generated in cell-free translation system using wheat germ extract, making their functionality in human cells an open question. In this vein, Ogawa et al. also report aptamer-mediated regulation of 3′ cap-independent translation elements which mediate alternative initiation mechanisms in plant viruses but are unsuitable for use in mammals [94]. However, IRES-mediated initiation is employed both by human-tropic viruses and human cells, suggesting that this strategy may be transferable [95]. IRES sequences have been used to promote expression in multiple AAV-delivered therapeutic systems, particularly those requiring multicistronic expression, making them an attractive regulatory target [96].

In addition to targeting alternative mechanisms of ribosome recruitment, Ogawa also reported riboswitch-mediated control of ribosomal shunting in a cell-free eukaryotic translation system [97,98]. Insertion of a split aptamer between a short upstream open reading frame (uORF) and a target downstream ORF (dORF) abolished reporter gene expression from the dORF. Aptamer binding promoted stem formation, allowing ribosomal shunting across the intervening aptamer and promoting dORF translation. Both the aptamer and uORF components are small and ribosome shunting is employed by viruses and human cells in several contexts including mediation of IRES activity, suggesting that this mechanism might be also be adapted for use in AAV-delivered transgene regulation [99,100].

### 2.4. Programmed Ribosomal Frameshifting Switches

-1 programmed ribosomal frameshifting (-1 PRF) describes a process in which the reading frame of an elongating ribosome is shifted 1 nt in the 5′ direction of an mRNA template [101]. Frameshifting occurs as the ribosome passes a UA-rich “slippery sequence” upstream of a stimulator structure, typically a pseudoknot. PRF enables a single locus to generate protein isoforms with different C-terminal sequences by encoding in multiple frames, but without bulky sequence elements such as introns or alternative exons. PRF is thus common in viruses, where genome space is at a premium, but also plays a role in both normal and disease-associated gene expression in humans [102]. In addition to promoting expression of alternative protein isoforms, -1 PRF can also mediate suppression of gene expression by shifting ribosomes into a frame with a premature stop codon [103].

Several groups have achieved small molecule-regulated -1 PRF by controlling stimulator formation using aptamers (Figure 2b). Chou et al. demonstrated that the hTPK pseudoknot found in human telomerase RNA could replace pseudoknot structures involved in -1 PRF, and that hTPK bore structural similarities to pseudoknot structures found in multiple bacterial riboswitches [104,105]. Replacement of an endogenous pseudoknot with a S-adenosylhomocysteine (SAH)-binding pseudoknot aptamer allowed 10-fold induction of -1 PRF in vitro, with further improvements made by RNA engineering and the clever use of adenosine-2′,3′-dialdehyde to inhibit SAH hydrolase [105]. Yu et al. pursued a similar strategy using pseudoknot-containing aptamers from several bacterial preQ1 riboswitches; a stabilized version of the *F. nucleatum* preQ1 aptamer could stimulate up to 40% of ribosomes to undergo -1 PRF in response to micromolar quantities of preQ1 [106]. Both of these systems were functional in reticulocyte lysates, pointing toward possible use in mammalian cells; however, only Chou et al. performed testing in human cells, where regulatory ranges were modest due in part to low cellular permeability to SAH.

Mechanistic studies of -1 PRF have shown that a 3′ hairpin (rather than pseudoknot) structure can also be used to regulate -1 PRF [107]. Noting a paucity of suitable pseudoknot-forming aptamers as well as regulation of terminator hairpin formation in bacterial riboswitches, Hsu et al. used both protein and theophylline aptamer-stabilized hairpins to regulate -1 PRF in HEK293 cells [108]. In contrast to stimulator pseudoknots, hairpin structures were placed upstream of the slippery sequence in these switches. Regulation could be further enhanced by replacement of the stimulator with a 3′ SAH aptamer-regulated pseudoknot: over 6-fold induction of -1 PRF was achieved in HEK293T cells using this dual-regulatory system. A later publication by this group reported novel stimulator sequences in which the theophylline aptamer controlled formation of a pseudoknot from SARS-CoV1 (SARS-PK) [109]. SARS-PK already serves as a stimulator of -1 PRF in mammalian cells during the course of SARS-CoV infection, making it a more relevant starting point for switch development compared to pseudoknot aptamers derived from bacterial riboswitches. The authors demonstrate synergy between these new theophylline-dependent 3′ stimulators with the previously-reported 5′ theophylline-dependent hairpin attenuators, and note that both mechanisms can now be regulated by a single small molecule. However, high (>100 µM) concentrations of theophylline are required due to lowered affinity after integration of the aptamer into the SARS-PK, and regulatory ranges in both reticulocyte lysates and HEK293 cells were lower than those obtained using their previously-developed switch. Matsumoto et al. report a higher dynamic range for their system, which also incorporates a -1 PRF system from a virus which infects mammals [110]. In contrast to the previous groups, these authors used a rationally-designed small-molecule ligand NCT8, which they had previously developed to mediate mismatch base pairing and loop-loop interactions [111]. The resulting switches enabled up to 9.1-fold induction of -1 PRF in HeLa cells; however, NCT8 is not approved for clinical use and lowered cell viability, placing it at a disadvantage for therapeutic applications compared to other small-molecule riboswitch regulators.

### 2.5. RNA Interference-Based Riboswitches

**Figure 3:**
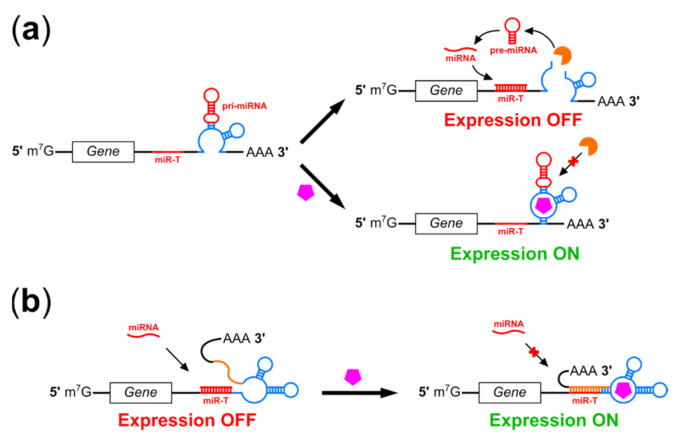
RNAi-Mediated Riboswitches in Mammalian Cells. (**a**) Regulation of miRNA processing. Pri-miRNA hairpins (red) can be conjugated to aptamers (blue) without disrupting processing, but ligand binding (magenta) occludes Drosha (orange). Insertion of these switches and an upstream miRNA target site (miR-T, red) into the 3′ UTR of an mRNA results in mRNA degradation through cleavage by Drosha, as well as silencing by the resulting miRNAs. Ligand binding blocks Drosha processing, inhibiting both cleavage mechanisms and restoring gene expression [120,121]. (**b**) Regulation of miRNA target site accessibility. Insertion of a tetracycline aptamer (blue) and short competing strand (orange) downstream of a miRNA target site (miR-T, red) allows tetracycline-mediated annealing of the competing strand to block RNAi and induce gene expression [125].

RNA interference (RNAi) is widely used in the laboratory to control transgene expression, and 2018 saw the first approval of an RNAi-based therapeutic in the United States [112,113]. RNAi involves hybridization of a short, complementary RNA or modified oligonucleotide to mRNA, triggering mRNA cleavage by the RNA-induced silencing complex (RISC). Interfering RNAs can be exogenous (siRNA, shRNA) or endogenous (miRNA), with miRNA processing requiring nuclear export and sequential cleavage by the enzymes Drosha and Dicer. miRNA target sequences are approximately 22 nt and miRNA expression cassettes are quite short, making it possible to deliver both RNAi and transgene expression cassettes in a single AAV; one recently published method for AAV delivery of RNAi expression cassettes advised the use of non-functional “stuffer” sequences in order to ensure that genomes could reach an adequate length for packaging into virions [114]. Aptamers may be used to regulate both the processing of engineered interfering RNAs delivered alongside a therapeutic transgene, as well as the accessibility of tissue-specific miRNA target sites, making RNAi-based riboswitches an attractive candidate for regulating AAV-delivered transgenes (Figure 3).

Some riboswitches function in mammalian cells using RNAi-based mechanisms but are not suitable for use with AAV vectors. Atanasov et al. replaced the terminal loop of a miRNA with an aptamer to the bacterial tet repressor (TetR) protein to generate tetracycline off-switches where binding of tetracycline to TetR prevents binding to the aptamer, thus promoting tet-mediated miRNA processing and activity [115]. In addition to displaying only 3-fold regulatory ranges these aptamers also require expression of the TetR protein, limiting vector headspace and possibly stimulating immune responses to TetR. Lin et al. developed a switch based on cell type-specific miRNA expression to enable specific killing of hepatocellular carcinoma cells. However, its length (7.3 kb) precludes use in AAV vectors [116]. A similar system developed by Matsuura et al. enabled more complex regulation of transgene expression by multiple miRNAs, but was also too large for AAV [117]. In addition to size constraints these latter two systems also require expression of the bacterial L7Ae RNA-binding protein, contributing to their larger sizes and also a risk of an immune response to the regulator protein. They also respond to miRNA rather than small molecule ligands, limiting regulatory strategies.

Several groups have reported RNAi-based riboswitches more suitable for regulating AAV-delivered transgenes. In 2006, An et al. reported a switch incorporating the theophylline aptamer in the loop region of an shRNA, demonstrating theophylline-mediated inhibition of miRNA processing and induction of reporter gene expression in HEK293 cells [118]. This strategy was further developed by Beisel et al., who used a thermodynamic model to design shRNA processing switches in silico [119]. These switches incorporated a competing strand to produce more significant structural rearrangements upon ligand binding and employed aptamers to tetracycline and hypoxanthine in addition to theophylline. Subsequent work by this group relocated the aptamer to the basal region of a pri-miRNA and inserted the resulting motifs into the 3′ UTR of a reporter transgene [120,121]. Addition of the switch ligand thus prevented both mRNA cleavage and release of a cis-acting pre-miRNA by Drosha without the need for a separate promoter for miRNA expression (Figure 3a). In contrast, Kumar et al. developed an RNAi-based off-switch using an allosteric ribozyme in which theophylline binding promoted self-cleavage and release of a functional pri-miRNA [122]. While all of these systems functioned in HEK293 cells, regulatory ranges were modest (~3 to 5-fold induction or suppression of reporter gene expression in response to 1.5–10 mM theophylline).

Despite limited dynamic ranges, a publication by Wong et al. demonstrates that careful selection of regulatory targets can enable highly effective regulation of mammalian cell behavior by RNAi-mediated riboswitches [123]. The authors modified the Beisel et al. switch to incorporate an aptamer to the chemotherapy drug folinic acid and placed multiple copies in the 3′ UTR of genes encoding cytokines, enabling up to 100-fold regulation of human T cell proliferation. A recent publication by Pollak et al. also used this system to regulate expression of cytochrome P450 1A2 (CYP1A2) in response to theophylline in HEK293 cells, achieving 5.7-fold induction of CYP1A2 expression [124]. The authors suggest that theophylline-induced expression of a detoxifying enzyme may be useful in reducing theophylline toxicity during emergency asthma treatment.

In addition to regulation of miRNA or shRNA processing, riboswitches have also been designed to use aptamer binding to control accessibility of a miRNA target site (Figure 3b). Mou et al. used the tetracycline aptamer to control annealing of a short competing strand to a miRNA target site, blocking miRNA targeting and enabling tetracycline-induced on-switch activity [125]. These switches showed up to 19-fold induction of gene expression, outperforming switches regulating miRNA processing. When blocking accessibility of endogenous miRNAs, the dynamic range of this system depended on miRNA expression levels; these switches could also regulate transgene expression in response to miRNAs delivered alongside the transgene in a single AAV vector. However, out of several candidate aptamers, only the tetracycline aptamer was used successfully, suggesting that this system may not possess the modularity observed in switches which regulate miRNA processing. Nonetheless, it may be possible to achieve improved dynamic ranges in a combined system in which tetracycline restricts Drosha cleavage and miRNA release from the 3′ UTR of a target gene, as well as accessibility of a miRNA target site within that gene.

### 2.6. Catalytic Riboswitches

**Figure 4:**
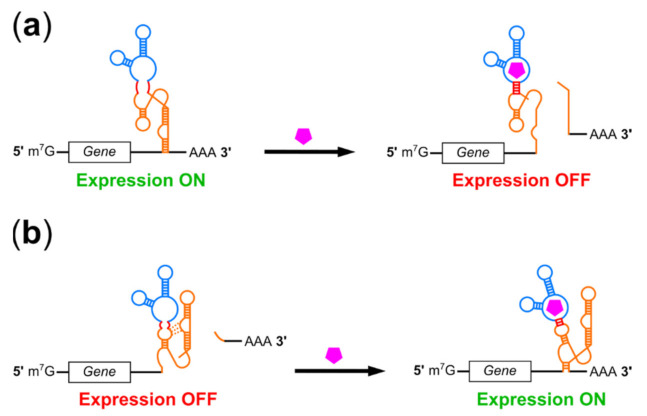
Aptazyme Riboswitches. (**a**) Aptazyme off-switches. An aptazyme consists of a self-cleaving ribozyme (orange) connected to an aptamer (blue) by a short CM (red). Ribozyme domains are inactive in the absence of ligand. Ligand binding (magenta) promotes CM annealing, which activates ribozyme cleavage and suppresses expression [130,131,137]. (**b**) Aptazyme on-switches. Self-cleavage is constitutively active in aptazyme on-switches, and is inhibited by ligand binding to promote gene expression [131,133,136].

Catalytic riboswitches comprise a rapidly-expanding set of riboswitches which most commonly modulate RNA stability using ligand-responsive, self-cleaving ribozymes, although ribozymes with other functions may also be used [23,75]. Self-cleaving ribozymes can mediate ligand-dependent removal of translation-essential motifs such as the 5′ m^7^G cap or poly-A tail while simultaneously exposing unprotected ends to exonuclease digestion (Figure 4). Although self-cleaving ribozymes can operate in reverse to act as RNA ligases, ribozymes have been optimized to retard the reverse reaction and cleavage can provide more permanent suppression of translation compared to mechanisms which rely on strand displacement or steric hindrance for sequestration of processing sites or blockage of the translation machinery [126]. This is reflected in the high dynamic ranges achieved by some aptazymes in mammalian cells (Table 1). Catalytic riboswitches can be classified based on their ligands: aptazyme riboswitches use a ligand-sensing aptamer domain to control ribozyme function, while other catalytic riboswitches employ RNA-binding proteins or complementary oligonucleotides as regulator molecules.

Aptazyme riboswitches were first described in 1997 by Tang and Breaker, who joined an ATP-binding aptamer to stem II of a self-cleaving hammerhead ribozyme using a short communication module (CM) and demonstrated ligand-dependent cleavage in vitro [29]. Follow-up work showed that aptazymes responsive to other ligands could be isolated by in vitro selection from libraries containing aptamers joined to the ribozyme by randomized CMs [40], with in vitro selected aptazymes capable of controlling transgene expression in bacteria and yeast [127,128]. In 2004, Winkler et al. reported a natural aptazyme switch which mediated feedback inhibition of the *glmS* gene in *B. subtilis* [129]. As with other types of bacterial or in vitro designed riboswitches, many of these aptazymes functioned poorly in mammalian cells. However, some bacterial aptazymes could be adapted to the mammalian cell environment through rational design. Taking a theophylline aptazyme which functioned in bacteria as a starting template, the Hartig group removed alternative start codons and optimized the CM sequence to achieve 6-fold suppression of reporter gene expression in HeLa cells treated with theophylline [130]. Meanwhile, the Smolke group adapted tetracycline- and theophylline-responsive aptazymes originally developed in yeast for use in human cells [131]. By placing tandem switches into the 3′ UTR of a cleavable reporter-cytokine fusion protein, the authors achieved theophylline-regulated T-cell proliferation in mice and in cultured human primary T lymphocytes; however, as with RNAi-based riboswitch control of T cell proliferation, the choice of a potent cell signaling molecule as a regulatory target likely helped amplify this switch’s regulatory range [123].

Aptazymes are versatile switches which may be used both to induce and to suppress transgene expression. For aptazyme off-switches, ligand binding promotes ribozyme activity and thus mRNA cleavage and degradation (Figure 4a), while in aptazyme on-switches, ligand binding suppresses self-cleavage and promotes expression (Figure 4b). Aptazyme on-switches face unique challenges compared to off-switches. On-switch ligands must bind and inhibit ribozyme activity immediately following transcription while off-switch ligands can bind at any point between transcription and translation. Furthermore ligand binding to on-switch aptamer domains must either remain bound for long timescales or promote lasting structural changes to inhibit cleavage, while off-switches require only transient ligand binding to activate it. Nonetheless, several aptazyme on-switches have been reported. Switches developed by Kobori et al. rely upon ligand-mediated ribozyme unfolding by an adjacent aptamer and were non-functional in mammalian cells despite attempts to optimize the expression platform [132]; however, a follow-up publication by Mustafina et al. used a similar mechanism to achieve over 6-fold activation of expression in mammalian cells in response to guanine [133]. Other aptazyme on-switches employ a more typical architecture in which aptamers are fused directly to helical stems within the ribozyme. Doxycycline-inhibited aptazyme on-switches were isolated by Piganeau et al. using in vitro selection of hammerhead ribozyme libraries bearing randomized stem II loop and stem I bulge regions [134]. It is worth noting that doxycycline binding by these switches requires sequence elements in both stems, predicting strategies for switching-capable aptamers based on selective randomization of RNA “scaffolds” [135]; however, to our knowledge, these devices have not been demonstrated to function in cells. The previously-mentioned switches developed by the Smolke group did function in human cells, but only displayed approximately 4-fold induction of reporter gene expression [131]. A more recent publication by Bielstein et al. reported aptazymes which inhibited hammerhead ribozyme activity in response to tetracycline, demonstrating 8.7-fold induction of gene expression in HeLa cells [136].

The well-studied hammerhead ribozyme is commonly used in catalytic riboswitches, but several other self-cleaving ribozymes have also been used as aptazyme expression platforms. Kertsburg and Soukup used a single CM to regulate hepatitis delta virus (HDV), hammerhead, X motif, and *Tetrahymena* group I intron ribozymes [137], and Beaudoin and Perreault attached a potassium-binding G-quadruplex motif to the HDV ribozyme to achieve K^+^-induced cleavage in vitro [138]. This latter group also demonstrated allosteric control of modified HDV ribozymes using RNA oligonucleotides, noting that extremely stable folding of the HDV ribozyme allowed it to perform well in a wide variety of conditions but also presented a challenge to strand invasion mechanisms of ribozyme inhibition [139]. More therapeutically-applicable HDV aptazymes were reported in 2013 by Nomura et al., who used the theophylline and guanine aptamers to control HDV ribozyme cleavage [140]. The authors generated libraries with randomized CMs joining either the theophylline or guanine aptamer to the HDV ribozyme and screened approximately 100 constructs from each library in human cells. Theophylline aptazymes exhibited modest (~4-fold) regulatory ranges, but the GuaM8HDV guanine aptazyme was able to suppress gene expression 29.5-fold in HEK293 cells and the switches could be combined to enable dual regulation. GuaM8HDV was later used by Strobel et al. to regulate transgene expression during AAV production; suppression of transgene expression in producer cells improved AAV yields by up to 23-fold [21]. It is worth noting here that transient suppression of transgene expression has also been used to improve yields during CAR-T cell production by preventing chimeric antigen receptor-mediated T cell fratricide [141]. This effect was demonstrated using the Tet-Off system; substitution of a riboswitch would prevent immune responses directed against Tet-Off protein components following CAR-T delivery. Because transgene suppression is performed on extracted T cells in vitro rather than in vivo, the superior performance of most riboswitches in cell culture compared to animal models makes their use here more feasible, although AAV-mediated CAR-T cell therapy has also been pursued [142]. GuaM8HDV performance in mice was compared to that of multiple hammerhead aptazymes by Reid et al., with the strongest control of reporter gene expression displayed by the tetracycline-responsive hammerhead aptazyme Tc45 [143]. This group also used Tc45 to control expression of the therapeutic VEGF inhibitor Eylea in a mouse model of age-related macular degeneration, showing tetracycline-mediated suppression of lesions associated with Eylea overexpression [143].

The HDV ribozyme evolved to function in the mammalian cell environment, but several groups have also attempted to adapt ribozymes from bacterial riboswitches for use in mammals. Kobori et al. selected aptazyme on-switches from libraries in which the *B. subtilis* guanine aptamer was placed upstream of a pistol ribozyme from *A. putredinis* and a stem region was randomized to promote mutually exclusive folding of either the aptamer or the ribozyme depending on ligand binding [132]. The authors found that the pistol ribozyme operated inefficiently in mammalian cells, and hypothesized that improving its function would allow construction of more efficient aptazymes. They subsequently screened approximately 3000 pistol variants in HEK293 cells using deep sequencing, and isolated several with improved function [144]. Felletti et al. have also adapted the bacterial twister ribozyme for use in eukaryotic cells, obtaining ligand-dependent expression control in yeast [145]. The authors noted that aptamers could be fused to two separate stems within twister simultaneously, and demonstrated complex expression control by aptazymes responsive to both theophylline and TPP. Mustafina et al. were able to adapt an on-switch which failed to function in mammalian cells by exchanging a pistol ribozyme for a twister ribozyme in the expression platform [132,133].

### 2.7. Improving the Function of Aptazyme Riboswitches

While their mechanism and modularity make aptazymes excellent candidates for transgene expression control, many exhibit modest (<10-fold) regulatory ranges. These compare poorly with other regulatory systems such as Tet-On and Tet-Off, which can activate or suppress transgene expression by up to three orders of magnitude in animal models [146]. This severely limits therapeutic applications and several methods have been pursued for improving the regulatory ranges of catalytic ribozymes in mammalian cells, as well as achieving suitable basal and suppressed/induced expression levels.

In addition to factors affecting the efficiency of non-catalytic riboswitches (e.g., ion concentration), aptazymes face the additional challenge of sequence- or organism-dependent effects on ribozyme catalytic efficiency [147], and non-allosteric ribozymes have been optimized for use in AAV-delivered gene therapy [148]. Efficient ribozyme domains improve aptazyme regulatory ranges by lowering basal expression in on-switches and enabling deeper suppression by off-switches, and several groups have optimized ribozymes specifically to improve catalytic riboswitch function. For example, to improve aptazyme switches, Yen et al. developed an optimized hammerhead ribozyme variant known as N107 which eliminated potential start codons and displayed almost ten-fold higher cleavage rates than its naturally-occurring parent construct [149]. N107-containing aptazymes were regulatable by aptamers binding adenosine and toyocamycin as well as by base pairing to complementary morpholino oligonucleotides, and several constructs exhibited small molecule-dependent gene regulation when delivered to mouse tissue using AAV. Zhong et al. further improved hammerhead ribozyme activity by limiting intra-ribozyme base pairing to promote dissociation after self-cleavage, lowering the rate of relegation and increasing the 18-fold suppression of transgene expression in HEK293T cells afforded by N107 to over 1000-fold [126]. Annealing of modified morpholino oligonucleotides complementary to the ribozyme resulted in 208-fold induction of luciferase expression in HEK293T cells, and 196-fold induction of a transgene encoding erythropoietin was achieved in mice by intramuscular injection of vivo-morpholinos [150]. These results represent some of the most efficient regulation of mammalian transgene expression without the use of exogenous proteins. However, modified oligonucleotide therapeutics are comparatively new and face additional regulatory and pharmacokinetic barriers for use as riboswitch regulators compared to the wide array of clinically-approved small-molecule drugs [151,152].

Optimizing the regulatory properties of an aptazyme more typically involves modifying switch placement within the mRNA, CM composition, and/or the relative orientations of aptamer and ribozyme motifs. Aptazymes are most frequently placed within the 3′ UTR of an mRNA to avoid inhibitory effects on translation, as switching elements are downstream of the stop codon but can still regulate expression through poly-A cleavage [127]. Kertsburg and Soukup demonstrated modest regulation of multiple expression platforms in vitro using a single, optimized CM [137], but maximizing an aptazyme’s regulatory range typically requires further CM tuning. Zhong et al. developed a rational design approach to improving CM function in tetracycline-regulated hammerhead aptazymes [153]. Beginning with a test panel of 32 aptazymes, the authors developed a scoring function for CMs which incorporated the number of hydrogen bonds, the proximity of base pairs to the ribozyme, and base stacking energies. This weighted hydrogen-bond and stacking score (WHSS) was highly predictive of aptazyme regulatory ranges and was used to develop additional aptazymes using the theophylline and guanine aptamers, as well as more efficient tetracycline aptazymes using aptamer stem P2 instead of stem P1 for CM attachment. This method required labor-intensive screening of dozens of constructs, but was quite successful; over 15-fold suppression of transgene expression was obtained in response to all three molecules in HeLa cells. One tetracycline aptazyme, Tc40, enabled over 20-fold suppression in human cells and also achieved 7-fold suppression of an AAV-delivered transgene through oral administration of tetracycline in a mouse model. Strobel et al. also recently demonstrated 15-fold induction of an AAV-delivered transgene in mice using a tetracycline-regulated aptazyme on-switch developed through a similar rational design and testing approach [154]. This result also represents a rare case in which switch performance was higher in an animal model than in previous results in cell culture [136]. A computational method has also been reported for developing protein-regulated aptazymes in silico [155].

Aptazymes may also be improved or generated by screening and/or selection of randomized libraries. Careful SELEX library design can enable selection of aptamer domains suited for regulating stem formation in switches, but these must be subsequently integrated into an expression platform and tested in cells [135]. Multiple strategies have been employed to screen and select entire allosteric ribozymes in cell-free systems [128,156,157,158,159,160,161]. However, while some in vitro selected aptazymes can function in human cells [162], many fail to operate outside of the selection environment [163]. Therefore, aptazymes have also been screened or selected within live bacteria [164,165,166] and yeast [48]. Bacteria and yeast are robust and easily-handled, but library sizes are limited by the transfection bottleneck [167] and riboswitches selected within them may also show reduced performance in mammalian cells. This difficulty is reflected in efforts to develop guanine-regulated aptazymes by Stifel et al., who enriched aptazymes using in vitro selection in *E. coli* but used rational design to develop less effective aptazymes for use in human cells [168]. Nonetheless, some aptazymes selected in prokaryotes or yeast can regulate transgene expression in mammals. Zhang et al. synthesized three theophylline aptazyme libraries with different architectures and randomized CMs, used FACS to select theophylline-responsive aptazymes in bacteria, and screened candidate switches in human and yeast cells [169]. The resulting switches could suppress reporter gene expression by 4.8-fold in HEK293T cells, and the authors demonstrated theophylline-regulated cell killing by ganciclovir in a model system similar to that used by Kim et al. [75]. Pu et al. also used bacterial cell selections to isolate aptazymes, which could control transgene expression in mammalian cells, albeit with lower regulatory ranges [170].

Selection within live mammalian cells would increase the probability that enriched aptazymes were functional in the target cell environment. However, in addition to more complicated cell culture techniques and limited library sizes, difficulties in introducing a single switch construct into each cell while still maintaining high transfection or transduction efficiency presents a challenge to aptazyme selections in mammalian cells. Several groups have used screening rather than selection to circumnavigate this difficulty. Xiang et al. transfected plasmids encoding barcoded theophylline aptazyme libraries into HEK293 cells, cultured them in the presence or absence of theophylline, and performed RNAseq to identify switch-containing mRNAs which showed differences in abundance in ligand-treated vs. untreated cells [171]. Results from the screen were validated using FACS-seq, and screens of additional libraries yielded aptazymes regulated by xanthine, folinic acid, and cyclic-di-GMP. Because relative mRNA abundance detected by sequencing was used to assay riboswitch performance, this method did not have the one-construct-one-cell requirement of selection methods involving cell separation and outgrowth based on reporter gene expression. Strobel et al. used a similar approach, screening libraries templated on tetracycline- and guanine-responsive hammerhead ribozymes and guanine-responsive HDV ribozymes in HEK293 cells using barcode-free deep sequencing to reduce library production costs [172]. This method identified previously-reported aptazymes as well as new functional variants. However, regulatory ranges were somewhat low for aptazymes identified by these screens compared to several rationally-designed switches.

In these screening methods, library sizes were constrained to ~10^5^ members by the detection limit of NGS; aptazyme candidates must be present in enough copies to obtain read depth sufficient to detect ligand-induced changes in abundance. In contrast selections enable the identification of RNA devices from much larger libraries, as functional switches are progressively enriched until they reach NGS detection thresholds. However, as mentioned previously, selecting large aptazyme libraries in cells is challenging. A recent publication by Townshend et al. presents a novel, automated method for selecting functional aptazymes from 10^12^–10^14^-member libraries followed by screening for function in live yeast [173]. In the DRIVER selection technique, iterative cleavage reactions in either the presence of the target ligand (positive selections) or structurally-similar small molecule decoys (negative selections) are performed in vitro. Both cleaved and uncleaved sequences are then regenerated using a method which also specifically labels cleaved vs. uncleaved sequences with separate priming sites, allowing specific reamplification of one or the other population for the next selection round. Automation reduces the time per round to approximately 3h, allowing hundreds of selection rounds to be performed. In aptazyme selections many cycles are required for enrichment due to slow removal of parasitic, non-switching sequences which can adopt both cleaving and non-cleaving conformations and thus achieve up to 50% survival in each round [157]. Enriched selection pools are next subjected to CleaveSeq screening where constructs are transferred into cells and then treated either with the target ligand or competitor molecules, followed by regeneration and screening for ligand-dependent cleavage using NGS. Several switches were also improved following DRIVER and CleaveSeq by mutagenic PCR and additional screening.

This selection and screening method is a powerful new tool for identifying not just novel aptazymes, but novel aptamers. By designing libraries with randomized regions in loops I and II of the hammerhead ribozyme the authors were able to select aptazymes responsive to five small molecules with no previously-reported aptamers, including a variant which produced 32.9-fold induction of transgene expression in yeast in response to the TLR7 agonist gardiquimod. Because randomized regions are inserted into separate loops, the selected ligand-binding domains may require engineering to operate as a compact ssRNA aptamer [134]. It is worth noting that Zhong et al. placed the aptamer domain on stem III in switches which performed well in mammalian cells; the base of stem III is immediately adjacent to the ribozyme cleavage site, possibly allowing more efficient regulation compared to modulation of stem II–stem I interactions [153,165]. However, selection of stem III libraries would be complicated because the cleavage fragment bearing the desired sequence information would not leave enough bases between the 3′ cleavage site and randomized stem III sequence for reverse priming during reamplification. Novel theophylline aptazymes selected using DRIVER and CleaveSeq showed lower regulatory ranges than previously-reported variants, suggesting that rational design or screening methods may be more effective for optimizing aptazymes using preexisting aptamers. Nonetheless, this system represents an exciting advance in aptazyme development; optimizing the in vitro selection environment and performing screening steps in mammalian cells might allow selection of aptazymes which regulate AAV-delivered transgene expression in response to highly-suitable ligands such as FDA-approved therapeutic small molecules with good bioavailability and few side effects.

### 2.8. Regulation of CRISPR-Cas Activity by Riboswitches

CRISPR-Cas systems represent powerful tools for gene therapy which enable targeted post-transcriptional expression control and genome editing, as well as a variety of other functions [174]. Despite size constraints CRISPR-Cas editing devices may be delivered using AAV vectors, where nuclear targeting of viral genomes can avoid immune responses to cytosolic DNA associated with other delivery mechanisms [175,176,177,178,179]. Aptamers have been used to recruit DNA modifying enzymes for base editing [180], to improve the efficiency and reduce off-target effects of HDR-mediated gene editing [181], and to target labeled CRISPR-Cas complexes to specific subcellular locations to improve imaging techniques [182], demonstrating that small, ligand-binding RNA devices can be integrated into CRISPR-Cas systems for a variety of purposes. For therapeutic applications, particularly gene editing, CRISPR-Cas systems must be tightly regulated both temporally and spatially. Other transgene regulatory techniques have been used to control guide RNA expression, but as previously discussed these systems have disadvantages for therapeutic applications [183]. Several groups have thus used riboswitches to regulate the activity of CRISPR-Cas.

**Figure 5:**
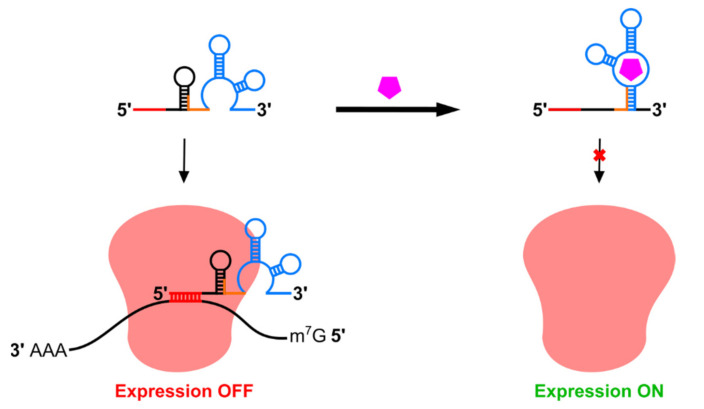
Regulation of CRISPR-Cas Single-Guide RNA Function. Single-guide RNAs (sgRNAs) are fusions of the multiple naturally-occurring gRNAs required for Cas protein targeting and activation [174]. sgRNAs contain a complementary region for targeting (red), along with several stem-loops onto which aptamers may be grafted to control folding (blue, orange). In the unbound state, sgRNAs are functional and can mediate mRNA degradation by Cas proteins (pink). Aptamer binding disrupts gRNA structure, blocking recruitment to Cas proteins and preventing mRNA cleavage. sgRNAs which are activated by aptamer binding have also been developed for use as off-switches [186,187,188].

In CRISPR-Cas systems, Cas effector proteins are targeted to specific nucleotide sequences using short-guide RNAs (gRNAs), including engineered single-guide RNAs (sgRNAs) which combine the multiple gRNAs of natural CRISPR-Cas systems into a single molecule [174]. Several groups have used aptamers to enable ligand-dependent control of CRISPR-Cas activity by regulating gRNA function (Figure 5). Kundert et al. used selection to develop gRNAs which could activate or repress CRISPR-Cas activity in bacteria in response to theophylline and 3-methylxanthine; however, these constructs were inactive in mammalian cells [184]. Iwasaki et al. also selected gRNAs bearing these two aptamers for function in bacterial cells, but did not demonstrate their function in eukaryotes [185]. Lin et al. generated gRNAs in which theophylline aptamer binding promoted refolding and Cas9 recruitment, and demonstrated modest (<1 fold) regulation of expression when these constructs were used in HEK293 cells [186]. Liu et al. used a strand displacement mechanism to control accessibility of the gRNA targeting region in response to tetracycline or theophylline, generating off- and on-switches which allowed complex dual regulation of CRISPR-Cas activity [187]. By using aptamers to two oncogenic proteins the authors were able to achieve specific killing of human cancer cells expressing both proteins despite low individual regulatory ranges. However, only one off-switch mechanism operated without the need for coexpressed viral proteins. Aptazyme riboswitches have also been used by Tang et al. to control gRNA function, enabling theophylline-induced genome editing and guanine-dependent targeting of transcriptional activators and achieving 5-6-fold regulation in each application [188]. Lin et al. recently used short trigger RNAs, including an endogenous miRNA, to modulate gRNA function in HEK293T cells, although as with aptazyme switches oligonucleotides are less favorable regulators than small molecules [189]. A particularly interesting case was recently reported by Renzl et al., who incorporated aptamers to the photoreceptor PAL into gRNAs and demonstrated 546-fold regulation of mRNA levels in response to light in HeLa cells [190]. Unfortunately the bacterial origin of PAL likely limit therapeutic applications of this system, but it represents an interesting optogenetic tool. In addition to controlling gRNA function, riboswitches have also been used to control expression of CRISPR-Cas effector proteins: Zhuang et al. used an aptazyme off-switch regulated by the cancer biomarker hTERT to control expression of Cas13d, achieving selective killing of hTERT-expressing bladder cancer cells [190].

**Table 1:** Performance of Small Molecule-Regulated Riboswitches in Mammalian Cells.

Switch Construct	Mechanism	Polarity	Ligand	Fold Regulation	Reference
A2	Polyadenylation	Off	Guanine	5.2	Spöring et al. 2020 [66]
L2	Splicing	On	Tetracycline	5.7	Vogel et al. 2018 [70]
Tet13_el	Splicing	On	Tetracycline	16.9	Finke et al. 2021 [71]
AS325 W-P9 6T8T	*Trans*-splicing	On	Theophylline	2	Kim et al. 2014 [75]
SVH2βgal	Translation roadblock	Off	Hoechst dyes	10	Werstuck and Green 1998 [79]
CFS-RX GAAA	Translation roadblock	Off	Ciprofloxacin	1.8	Groher et al. 2018 [85]
R22	Translation roadblock	Off	Theophylline	10	Liu et al. 2018 [89]
R26	Translation roadblock	Off	Tetracycline	10	Liu et al. 2018 [89]
68 *metH*	Programmed ribosomal frameshifting	On/Off	Adenosine-2’,3’-dialdehyde	4.39	Chou et al. 2010 [105]
Theo-OFF2-MMTV	Programmed ribosomal frameshifting	On/Off	Theophylline	6	Hsu et al. 2014 [108]
TheoOFF2 SARS	Programmed ribosomal frameshifting	On/Off	Theophylline	1.5	Lin and Chang 2016 [109]
M1-VPK	Programmed ribosomal frameshifting	On/Off	NCT8	9.1	Matsumoto et al. 2018 [110]
pE19T	miRNA processing	On	Theophylline	3	An et al. 2006 [118]
th1	miRNA processing	On	Theophylline	4.1	Beisel et al. 2011 [120]
pRzTheo-miREGFP	miRNA processing	Off	Theophylline	4	Kumar et al. 2009 [122]
β(1x)	miRNA processing	On	Folinic acid	3.5	Wong et al. 2018 [123]
miR-378a-CT	miRNA processing	On	Theophylline	5.7	Pollak et al. 2021 [124]
tac210	miRNA site accessibility	On	Tetracycline	19	Mou et al. 2018 [125]
P1-F5, 5.3	mRNA self-cleavage	Off	Theophylline	6	Ausländer et al. 2010 [130]
7c4x	mRNA self-cleavage	On	Guanine	6.7	Mustafina et al. 2020 [133]
K19	mRNA self-cleavage	On	Tetracycline	8.7	Beilstein et al. 2015 [136]
GuaM8HDV	mRNA self-cleavage	Off	Guanine	29.5	Nomura et al. 2013 [140]
Tc40	mRNA self-cleavage	Off	Tetracycline	24	Zhong et al. 2016 [153]
Gua_K3	mRNA self-cleavage	On	Guanine	4	Stifel et al. 2019 [168]
Theo-HHR-B	mRNA self-cleavage	Off	Theophylline	4.8	Zhang et al. 2017 [169]
TAP1	mRNA self-cleavage	Off	Theophylline	7	Pu et al. 2020 [170]
XanACGAG	mRNA self-cleavage	On	Hypoxanthine	6.8	Xiang et al. 2019 [171]
FolUGAAG	mRNA self-cleavage	On	(6R,S)-folinic acid	5.3	Xiang et al. 2019 [171]
cdG-CGUAA	mRNA self-cleavage	On	Cyclic di-GMP	2	Xiang et al. 2019 [171]
sgRNA4	CRISPR-Cas gRNA accessibility	Off	Tetracycline	4.25	Liu et al. 2016 [187]
Theophylline-agRNA	CRISPR-Cas gRNA cleavage	On/Off	Theophylline	3	Tang et al. 2017 [188]
Guanine-agRNA	CRISPR-Cas gRNA cleavage	On/Off	Guanine	4.8	Tang et al. 2017 [188]

### 2.9. Deoxyribozyme Switches

One of the advantages of AAV as a transgene delivery vehicle is its ability to provide long-term expression from DNA episomes in the target cell nucleus [191], but this can rapidly become a disadvantage if a patient experiences a deleterious response to the transgene product. Riboswitches which mediate mRNA processing, translation efficiency, or stability may not be suited to long-term suppression of a harmful transgene product based on basal/suppressed expression levels or the need to continuously supply high levels of a regulator ligand. It is thus desirable to obtain a “kill switch” which can mediate destruction of AAV genomes or episomes; this strategy has been implemented in self-deleting, AAV-delivered CRISPR-Cas systems, but a ligand-mediated kill switch would represent an easier, less-invasive method for episome removal in a broader range of AAV therapies [192,193]. The majority of reported riboswitches are composed of RNA and to our knowledge no naturally-occurring deoxyribozymes have been reported, although several have been isolated through in vitro selection. These include devices capable of cleaving DNA in cis and/or trans [194,195,196,197], as well as trans-cleavage of RNA [198,199]. Because DNA does not possess RNA’s 2′ hydroxyl group, deoxyribozymes cannot use the nucleophilic 2′-3′ cyclization mechanism employed by many ribozymes. Instead deoxyribozymes depend on metal ion cofactors, and several have been developed for use as metal ion biosensors [200]. Deoxyribozymes can function in human cells, and several therapeutic applications have been suggested [201,202]. In addition, allosteric deoxyribozymes can regulate catalytic activity in vitro in response to proteins and short nucleic acids [203,204,205]. Deoxyribozyme kill switches embedded in AAV genomes would require extremely low basal activity in order to maintain long-term episomal expression, and both proteins and trans-cleaving ribozymes would be easier to express. Nonetheless, their ability to function in human cells suggests that further work may yield new allosteric deoxyribozymes for use as small molecule-induced AAV “kill switches,” improving the safety of AAV-delivered gene therapy.

## 3. Therapeutic Applications of Riboswitches

The complexity of human biology and the wide array of human pathogens together yield an enormous variety of potential gene therapy targets, while AAV and oligonucleotide therapeutics have also expanded rapidly in the past decade [15,206]. This provides a wide array of possible uses for riboswitches in gene therapy [207]. Potential therapeutic applications have been demonstrated for many of the riboswitches discussed in the previous section, including regulation of T cell proliferation [123,131,141], regulation of VEGF-targeted therapeutics to prevent overdosing [145], control of targeted genome editing [188], selective killing of cancer cells [75], reducing the toxicity of asthma treatment [124], and improving production of therapeutic AAV [21]. This section will take a narrower focus, discussing the applicability of riboswitch-regulated, AAV-delivered transgene therapy to control dosing of a therapeutic signaling molecule and to improve safety of a pathogen defense strategy. These examples illustrate how riboswitches might be used to enhance several aspects of AAV-mediated transgene therapeutics.

### 3.1. Regulation of Erythropoeitin Expression

Erythropoietin (Epo) is a glycoprotein cytokine synthesized by the kidney and liver to regulate red blood cell proliferation, as well as to modulate other processes in the vascular and central nervous systems [208]. Epo is a well-studied therapeutic target implicated in anemia, erythrocytosis, and chronic renal failure. Recombinant rhEpo has been used therapeutically to treat anemia but production costs are high, and rhEpo administration has also been associated with hypertension and thrombosis. Long-term, AAV-mediated Epo expression has been achieved in animal models, promoting blood cell proliferation and neuroprotective effects [209,210,211]. However, expression control is highly desirable to enable maintenance of homeostatic red blood cell counts. Multiple expression control systems based on engineered proteins have been used for this purpose, but these systems have had trouble maintaining long-term controlled expression and adverse immune reactions have been observed against both vector components and Epo [212,213,214].

Several groups have pursued riboswitch regulation of Epo expression as an alternative, demonstrating impressive results in animal models. Zhong et al. achieved over 200-fold induction of an AAV-delivered transgene encoding Epo using morpholino-regulated hammerhead ribozymes, and morpholino injection could induce Epo production over 43 weeks after a single administration of AAV [126]. Hematocrit could be tuned to homeostatic levels through controlled dosing of morpholinos and stable, homeostatic Epo levels were maintained for weeks after a single morpholino injection. As discussed previously, small-molecule drugs are more attractive regulator candidates than oligonucleotides, a fact recognized by several groups developing Epo gene therapies: in 2008 the Mirus Bio Corporation applied for a small business innovation research (SBIR) grant for development of drug-sensing riboswitches for regulation of Epo expression [215]. More recently, patents were filed in multiple countries by Meiragtx UK Limited for an erythropoietin expression control system based on aptamer regulation of alternative splicing [216]. In addition to controlling expression, riboswitches may also help to prevent deleterious immune responses to transgenic erythropoietin, including development of anti-Epo autoimmunity [214]. Anti-transgene immune responses present a challenge to many gene therapies, and the use of riboswitches to address this problem in AAV-delivered gene therapy is discussed in further detail in the following section.

### 3.2. Regulation of Vectored Immunoprophylaxis

Vectored immunoprophylaxis (VIP) is a method by which transgenes encoding immune effectors (most commonly monoclonal antibodies or antibody derivatives) are expressed from a patient’s cells to prevent infection [217]. HIV is a common target for VIP because of the extreme rarity of effective neutralizing antibody development in patients; despite enormous efforts a successful HIV vaccine has yet to be developed [218]. Small molecule therapies have been extremely effective at reducing morbidity and mortality but require regular administration, can produce negative side effects, and are susceptible to escape mutations [219]. Administration of synthetic broadly-neutralizing monoclonal antibodies (bnAbs) can prevent infection and reduce viral titers, but bnAbs also must be regularly administered and their production is expensive and complicated [220]. VIP circumnavigates these difficulties through long-term bnAb expression, bypassing the need for endogenous bnAb development or frequent drug administration. VIP was first reported by Lewis et al. in 2003, who delivered an anti-HIV bnAb to mouse muscle tissue using AAV and observed HIV neutralization by sera up to 6 months after a single administration [221]. Subsequently VIP has also been demonstrated to provide protection in animal models from several other viruses [222,223,224,225,226,227], anthrax [228], and malaria [229]. AAV-mediated anti-HIV VIP was also the subject of a recent Phase I clinical trial in the UK, where the therapy was well tolerated but bnAb expression levels were often low and several patients developed anti-bnAb antibody responses [230].

Patients receiving VIP risk developing immune responses to both the AAV capsid and the engineered, non-self proteins used to target pathogens. Delivering therapies to the liver can promote a T cell-mediated reduction in anti-capsid and anti-transgene responses, but innate immune responses can still occur and VIP typically uses expression from muscle tissue [231,232]. While other AAV-delivered therapeutics can provoke dangerous immune responses such as anaphylaxis or autoimmunity [214,233], anti-transgene responses in VIP are more typically associated with reduced bnAb titers [217,234,235]. This, alongside a need for higher bnAb expression, means that on- and off-riboswitches may not be suited to long-term regulation in VIP. However, transient immunosuppression during AAV administration has been shown to reduce the occurrence of anti-transgene immunity and improve expression levels [217,236]. RNA off-switches could serve as a safer alternative to immunosuppression, allowing coadministration of AAV and an off-switch ligand which suppresses transgene expression until the heightened immune surveillance observed to follow vector administration has subsided [237]. In addition, the US Defense Advanced Research Projects Agency (DARPA) has developed the PREPARE program, which seeks to achieve inducible, transient expression of protective transgene products in military service members, first responders, and civilians [238]. One of the targets of this program is influenza infection; in addition, PREPARE is also pursuing inducible transgene-mediated protection from opioid overdose, organophosphate poisoning, and gamma radiation. RNA on-switches are attractive candidates for this purpose as their modularity would allow the use of multiple aptamers, enabling specific induction of one or more transgenes by different ligands.

## 4. Conclusions

Riboswitches, particularly aptazyme and RNAi switches, represent an attractive method for control of AAV-delivered therapeutic transgene expression due to their small sizes, non-immunogenicity, modular structures, and ability to function without protein switching elements. Expression control by riboswitches has been demonstrated in human cells and in animal models, allowing modulation of therapeutic protein levels and biological processes such as antibody expression and blood cell proliferation. Riboswitches may also help to improve other aspects of AAV therapy such as vector yields and anti-transgene immune responses. Aptazymes may also be incorporated into more complex AAV-delivered therapeutic systems such as CRISPR-Cas-mediated expression control and gene editing. However, the efficiency of regulation by riboswitches must be improved in the mammalian cell environment for many clinical applications. Although they have been applied in some other cell types such as T cells, many riboswitches are tested in either HEK293 or HeLa cells and riboswitch performance in different cell types merits further exploration. Furthermore, performance in animal models compares poorly to that in cultured cells, possibly due to immune effects or pharmacokinetic limitations of regulator molecules which do not apply in cell culture. However, recent advances in rational design and screening strategies have significantly improved the performance of multiple riboswitches, particularly aptazymes regulated by tetracycline, theophylline, or guanine. Meanwhile, a novel selection and screening strategy may enable rapid isolation of aptazymes which function in the mammalian cell environment and respond to novel, high-performance small-molecule ligands without the need for preexisting aptamers or SELEX. Taken together, these results show that riboswitches are increasingly potent regulators of gene expression in mammals which comprise a versatile, rapidly-expanding toolset for expression control in AAV gene therapy.

## Data Availability

No new data were created or analyzed in this study. Data sharing is not applicable to this article.
