# Correction: Tickner, Z.J.; Farzan, M. Riboswitches for Controlled Expression of Therapeutic Transgenes Delivered by Adeno-Associated Viral Vectors. *Pharmaceuticals* 2021, *14*, 554

**DOI:** 10.3390/ph14121271

**Published:** 2021-12-06

**Authors:** Zachary J. Tickner, Michael Farzan

**Affiliations:** 1 Department of Immunology and Microbiology, The Scripps Research Institute, Jupiter, FL 33458, USA; mfarzan@scripps.edu; 2 Emmune, Inc., Jupiter, FL 33458, USA

## Missing Citation

In the original article, Renzl, C.; Kakoti, A.; Mayer, G. Aptamer-Mediated Reversible Transactivation of Gene Expression by Light. *Angew. Chem. Int. Ed.* **2020**, *59*, 22414, doi:10.1002/anie.202009240 was not cited [1]. The citation has now been inserted in Section 2.8: *Regulation of CRISPR-Cas Activity by Riboswitches,* paragraph 2, and should read: A particularly interesting case was recently reported by Renzl et al., who incorporated aptamers to the photoreceptor PAL into gRNAs and demonstrated 546-fold regulation of mRNA levels in response to light in HeLa cells [190].
